# Small RNA Profiling of Two Important Cultivars of Banana and Overexpression of miRNA156 in Transgenic Banana Plants

**DOI:** 10.1371/journal.pone.0127179

**Published:** 2015-05-11

**Authors:** Siddhesh B. Ghag, Upendra K. S. Shekhawat, Thumballi R. Ganapathi

**Affiliations:** Plant Cell Culture Technology section, Nuclear Agriculture & Biotechnology Division, Bhabha Atomic Research Centre, Mumbai, Maharashtra, India; Kunming University of Science and Technology, CHINA

## Abstract

Micro RNAs (miRNAs) are a class of non-coding, short RNAs having important roles in regulation of gene expression. Although plant miRNAs have been studied in detail in some model plants, less is known about these miRNAs in important fruit plants like banana. miRNAs have pivotal roles in plant growth and development, and in responses to diverse biotic and abiotic stress stimuli. Here, we have analyzed the small RNA expression profiles of two different economically significant banana cultivars by using high-throughput sequencing technology. We identified a total of 170 and 244 miRNAs in the two libraries respectively derived from cv. *Grand Naine* and cv. *Rasthali* leaves. In addition, several cultivar specific microRNAs along with their putative target transcripts were also detected in our studies. To validate our findings regarding the small RNA profiles, we also undertook overexpression of a common microRNA, *MusamiRNA156* in transgenic banana plants. The transgenic plants overexpressing the stem-loop sequence derived from *MusamiRNA156* gene were stunted in their growth together with peculiar changes in leaf anatomy. These results provide a foundation for further investigations into important physiological and metabolic pathways operational in banana in general and cultivar specific traits in particular.

## Introduction

A large number of small RNAs have been identified in plants which play significant roles in the genetic regulation at the post transcriptional level [1, 2]. These small RNAs, majority of them comprising the members of micro RNA (miRNA) family are transcribed as primary-miRNA transcripts by RNA polymerase II. These are then processed by dicer-like 1 (DCL1) proteins to ultimately form single stranded non coding 21–24 nucleotide molecules that get incorporated into the RNA Induced Silencing Complex (RISC). The single stranded small RNA guides the RISC complex to the cognate mRNA transcript and cleaves it bringing about sequence specific post transcriptional gene silencing [3]. Huge amount of data accumulated in the last decade proves the involvement of small RNAs in growth, development, abiotic and biotic stresses regulation in plant [4]. Studies demonstrating the vital role of small RNAs in model plants have been carried out extensively but the easy availability of high throughput sequencing technologies have now made this possible in other economically important crop plants also. Small RNA profiling has been attempted previously in rice [5, 6], tomato [7], brassica [8], mulberry [9], potato [10] and maize [11] to study the conserved and novel families of miRNA in different cultivars of these plants.

Banana is the fourth most important crop after the major cereal crops with a world production of 1.1 billion metric tonnes per year (http://www.statista.com/statistics/264001/worldwide-production-of-fruit-by-variety/). Bananas are grown in more than 120 countries across the globe and are regarded as an important food security crop. There are more than 50 different cultivars grown in the Indian subcontinent each bearing unique fruit characteristics [12]. We have performed small RNA profiling of two commercially important dessert cultivars of banana—*Grand Naine* (AAA subgroup Cavendish) and *Rasthali* (AAB subgroup Silk).

*Grand Naine* is one of the most commonly cultivated commercial Cavendish cultivar in the world. It is a relatively tall banana plant with long bunch bearing 200–220 fruits. The fruits are yellowish green, delicious to eat and have good keeping quality. *Rasthali* is cultivated mostly in India and is popular in local and world market as a premium dessert variety similar to Cavendish bananas [13]. The fruit is medium sized with ivory yellow colour flesh within a thin skin. It has a unique sweet acidic taste [14]. Cultivar *Rasthali* is highly susceptible to Fusarium wilt disease whereas *Grand Naine* is resistant to race 1 of *Fusarium oxysporum* f. sp. *cubense* (the causal agent of Fusarium wilt of banana). The recent unearthing of the banana genome [15, 16] has led to renewed interest in performing genome wide studies to understand the variability associated with disease resistance, fruit characteristics and environmental stress tolerance. In this study, we have carried out small RNA sequencing of two cultivars of banana belonging to two different subgroups to determine the difference in the quantum of common miRNAs and identify the novel genome specific miRNAs in the two samples.

Among the thoroughly studied miRNAs in model plants is the miRNA156 which is highly conserved and expressed mostly in young plant parts and play important role in plant growth and development [17]. miRNA156 expression is found to be high during juvenile stage of plant growth and it later decreases as the plant matures [17]. Overexpression of miRNA156 in Arabidopsis, maize and rice results in gross morphological changes in the plant. miRNA156 targets the Squamosa Promoter binding protein Like (SPL) family of genes having at least one Squamosa Promoter Binding (SBP) domain. These proteins regulate the expression of SPL proteins involved in plant development such as phase transition [18], flowering pattern [19], plant fertility [20] and secondary metabolite production [21]. The role played by miRNA156 in early plant growth and the difference in the phenotypic characteristics of the two banana varieties under study compelled us to investigate its role in banana. Thus as an extension of small RNA sequencing study, we overexpressed native miRNA156 gene in transgenic banana plants to establish its importance in banana growth and development.

## Materials and Methods

### Plant materials

Suckers were obtained from the two banana (*Musa* spp.) cultivars namely *Grand Naine* (subgroup: Cavendish) and *Rasthali* (subgroup: Silk) having triploid genome composition and belonging to AAA and AAB group respectively. The suckers were grown under green house conditions. Tissue from the fresh fully expanded leaves was collected and used for small RNA isolation.

### Isolation of small RNA from banana leaf tissue and Illumina sequencing

Total RNA was extracted from the fully expanded fresh banana leaves using Concert Plant RNA Reagent (Invitrogen, USA). The samples were incubated for 5 min at room temperature before separation of the supernatant. The supernatant was treated with 5M sodium chloride and the aqueous phase was extracted using chloroform. The chloroform extract was precipitated by adding MgCl_2_ (10 mM) and 2.5 volumes of chilled absolute ethanol. The samples were incubated at -20°C for 30 minutes and centrifuged at 16000 *g* for 20 minutes at 4°C to pellet down the total RNA. Total RNA obtained was dissolved in 45 μl RNase free water at 65°C. Further, the small RNA fraction was isolated from the total RNA using *mir*Vana miRNA Isolation Kit (Ambion, USA) following the manufacturers instructions. Small RNA bound to the column was eluted in 40 μl RNase free water). Small RNA was separated based on the size using denaturing gel. Later the 5’ and 3’ adapters were ligated to the small RNAs and reverse transcribed and PCR amplified. Quality and quantity of the RNA was determined by using Bioanalyzer. The library construction and sequencing was performed at Genotypic Technology (P) Ltd (Bangalore, India) on the Illumina GAIIX platform.

### Bioinformatic analysis banana miRNAs

After the adapter sequences were trimmed, sequences with low quality or length less than 15 nucleotides were removed. The small RNA sequences obtained were compared with the miRBase database [22] and the NCBI nucleotide database to remove housekeeping small RNAs like tRNA, rRNA and small nucleolar RNA. Banana genome sequence database available at http://banana-genome.cirad.fr/ was used to analyze the genomic origins of the remaining small RNAs and subsequently small RNAs unique to each cultivar and similar in both the cultivars were identified.

### Target prediction

For prediction of miRNA targets of the identified conserved and predicted novel miRNAs, miRanda target prediction tool was used. miRNA sequences were fed as input along with *Musa acuminata* mRNA sequences using the “strict” settings (Require strict alignment in the seed region (offset positions 2–8). This option prevents the detection of target sites which contain gaps or non-canonical base pairing in this region). miRNA hits having minimum free energy < = -40 are assumed to be most probable targets for reported miRNA.

### Accession Numbers

Small RNA sequence data derived from *Rasthali* and *Grand Naine* have been deposited at the National Centre for Biotechnology Information Gene Expression Omnibus archive under the Bioproject PRJNA274803 (accession numbers GSE65728 and GSE65727 respectively).

### Isolation and cloning of miRNA156

Precursor miRNA (pre-miRNA) sequences comprising the stem loop of *OsmiR156a* (accession no.: MI0000653) from rice was used as template to identify similar *pre*-*miR156* sequences in the banana genome database. Several partial hits were examined *in silico*. These sequences were analyzed for their capacity to fold into typical miRNA hairpins. Homolog of one of these sequences was found in the EST database of banana (accession no.: ES434836). This sequence was aligned with the genomic sequence derived from *Musa acuminata* subspecies *malaccensis* (genomic location: GSMUA_Achr7G05222_001) sourced from banana genome hub and primers were designed in the complementary regions. A 175 bp sequence encompassing the stem loop sequence of miRNA156 gene was subsequently amplified from genomic DNA derived from banana cv. *Rasthali* leaves using these primers. This sequence was first cloned into pTZ57R/T cloning vector and then subsequently subcloned in p1301-*MusaDAD1* ^14^ in place of *MusaDAD1* coding sequence to generate p1301-*MusamiRNA156* binary vector. This construct was electroporated into *Agrobacterium tumefaciens* strain EHA105 for use in banana genetic transformation.

### Generation of transgenic banana plants

The transgenic banana plants were generated by *Agrobacterium*-mediated genetic transformation method as described previously [23, 24]. The *Agrobacterium* culture harbouring p1301-*MusamiRNA156 binary* vector was cocultivated with embryogenic cell suspension cultures of banana cv. *Rasthali*. The cocultivated banana cells were aspirated onto the glass fibre filters and placed on the medium supplemented with acetosynringone (ACS). After 3 days the cells along with the filters were transferred onto medium containing cefotaxime (400 mg/L) and incubated for another 3 days. On the seventh day post cocultivation the cells were transferred onto medium supplemented with cefotaxime (400 mg/L) and hygromycin (5 mg/L). The embryos developed on the hygromycin based medium were subcultured for three rounds after every 3 weeks. The embryos were germinated in medium containing 0.5 mg/L 6-benzylaminopurine (BAP). The shoots were multiplied in medium containing BAP and rooted in medium added with 1-naphthalene acetic acid (NAA). The rooted plantlets were hardened in green house under controlled conditions.

### Histochemical GUS staining

The GUS activity in the transgenic banana plants overexpressing *MusamiRNA156* was detected by histochemical staining with 5-bromo-4-chloro-3-indolyl-β-D-glucuronic acid (X-Gluc). The leaf tissue sample from transgenic banana plant and untransformed control was incubated overnight at 37°C in staining buffer (1 mM X-Gluc, 100 mM sodium phosphate (pH 7.0), 10 mM EDTA, 0.5 mM K_4_Fe(CN)_6_, 0.5 mM K_3_Fe(CN)_6_, and 0.1% (v/v) Triton X-100) as described previously [25]. Following day the samples were fixed in 70% ethanol and photographed.

### Detection of precursor miRNA156 sequence by RT-PCR

Total RNA was extracted from the leaf tissue of a selected transgenic banana plant and an untransformed control banana plant as described above. cDNA was synthesized using oligo (dT)_12–18_ primer and AccuScript Reverse Transcriptase (Invitrogen, USA). The cDNA preparation was diluted appropriately before being used for PCR amplification. PCR was carried out using the following primers: *miRNA156* Fw: AGGAGATCGGCGACGGATA *miRNA156* Rv: GGGGGTTGACAGAAGAGAGTG. Following amplification protocol was performed: 94°C for 5 min followed by 35 cycles each with 94°C for 1 min, 56°C for 1 min and 72°C for 45 sec with a final extension 72°C for 5 min. The amplified products were separated on 1.5% (w/v) TAE-Agarose gel and visualized using ethidium bromide.

### Morphological analysis

Transgenic banana plants along with the untransformed control banana plants were hardened together in the green house under controlled conditions. The plants were observed for change in the height, leaf anatomy and root growth after 1 month. The plants were further maintained in green house and observed for morphology after 3 and 6 months period. The abaxial surface of the leaves was observed under microscope after 1 month to study the change in the leaf cellular arrangement.

## Results

### High throughput sequencing of miRNAs in banana cultivars

**Fig 1:**
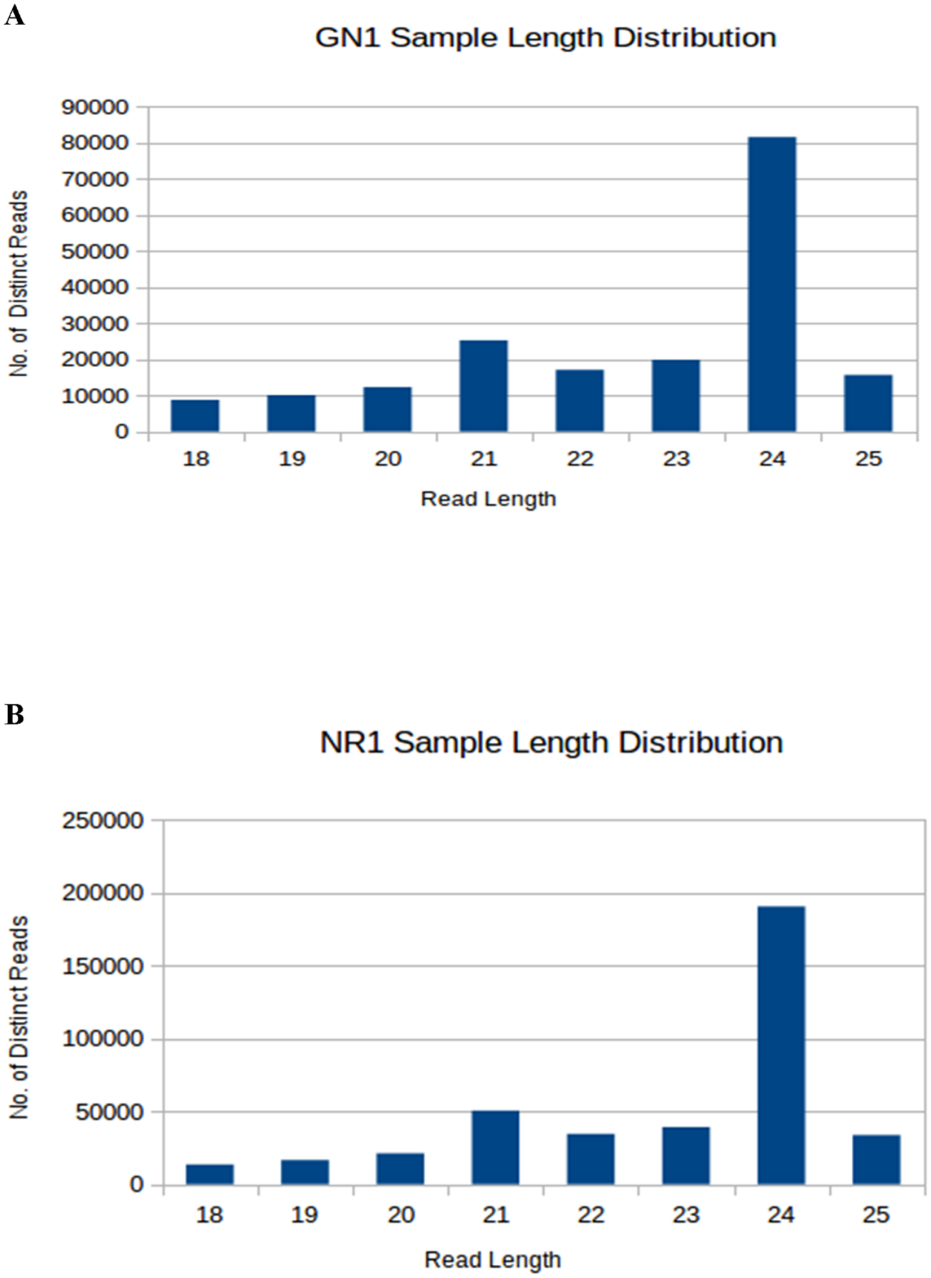
Size distribution of small RNA sequences. Sequence reads and unique sequence distribution showing the predominance of the 24 and 21nt length reads in *Grand Naine* (A) and *Rasthali* (B) small RNA libraries.

To investigate the difference in the small RNA profiles with regard to their quantum and identity in two cultivars of banana—*Grand Naine* (GN1) and *Rasthali* (NR1), belonging to subgroups Cavendish (AAA) and Silk (AAB) respectively, small RNA sequencing was carried out using Illumina sequencing platform. A total of 4981328 and 8889596 reads were obtained from *Grand Naine* and *Rasthali* small RNA libraries respectively (Table 1). A total of 190680 and 399953 sequences were filtered out after 3’ adaptor removal and length range filtering. Among these the most abundant small RNAs were 24-nt long followed by 21 nt long sequences (Fig 1).

**Table 1:** Summary of the total and distinct number of reads of *Grand Naine* (GN1) and *Rasthali* (NR1) libraries.

	Grand Naine (GN1)		Rasthali (NR1)	
	Total reads	Distinct reads	Total reads	Distinct reads
Total number of sequences (reads) in input file	4981328	567079	8889596	1054705
Total number of sequences remaining after 3' adaptor removal	3298656	410366	3475517	527435
Total number of sequences remaining after length range filtering (18–25)	905382	190680	2298121	399953

### Identification of conserved and novel miRNAs

For identification of conserved miRNAs in the two banana cultivars, the small RNA sequences obtained were compared against known mature plant miRNA sequences deposited in the miRBase database. A total of 127and 163 unique miRNA sequences were identified in *Grand Naine* and *Rasthali* cultivars (Table 2). These comprised 55 and 71 families of microRNAs respectively in *Grand Naine* and *Rasthali* cultivars (S1 Table). Most of these miRNAs displayed good sequence conservation with miRNAs identified in other plants. To identify novel miRNA sequences in our libraries, reads which were filtered in after 3’ adaptor removal and length range filtering and which did not align to mirBase were analysed using Mireap along with banana genome (*Musa acuminata*) database. Mireap identified novel microRNA based on alignment, secondary structure, free energy and location on the precursor arm. 55% of reads that did not map to mirbase were successfully mapped on to banana genome in *Rasthali* whereas this number was a close 56% in case of *Grand Naine*. We predicted 81 novel microRNAs in *Rasthali* and 43 in *Grand Naine* (Table 3; S2 Table).

**Table 2:** Summary of miRNAs identified in the *Grand Naine* and *Rasthali* libraries.

	Grand Naine (GN1)	Rasthali (NR1)
miRNA considered	Plants (ViridiPlantae)	
Total number of predicted miRNA	17986	28095
Total number of distinct miRNA	127	163
miRNA having number of reads >10	48	63
Unique miRNA (present only in *Grand Naine*/ *Rasthali* sample)	55	91
Unique miRNA (present only in *Grand Naine*/ *Rasthali* sample) >10	7	10
Common miRNA (present in both samples)	72	

**Table 3:** Statistics of the novel miRNAs in the two libraries used for target prediction.

Small RNAs [18-26nt]	Grand Naine (GN1)	Rasthali (NR1)
Total Reads	4981328	8889596
Total reads that did not map to mirBase used for novel miRNA prediction	188621	396431
Total Reads Mapped to genome (*Musa acuminata*)	105799	217931
Total number of novel miRNA identified	43	81
Total number of novel miRNA's taken for target prediction (> = 10 Read count cut-off)	16	20

### Identification of cultivar specific miRNAs

Owing to the fact that the cultivars analysed belonged to different subgroups of banana, we also tried to identify miRNAs which were specific to each cultivar and those which are common to both of them. Out of a total of 163 and 127 miRNA sequences identified in *Rasthali* and *Grand Naine* cultivars as mentioned above, 72 were common among the two whereas 91 and 55 miRNAs were respectively unique to *Rasthali* and *Grand Naine* (S1 Table).

### Target prediction

Since plant miRNAs possess perfect or near-perfect complementarity to their respective target-site, usage of computational tools allows effective prediction of the target sequences. To identify putative targets of conserved and cultivar specific miRNAs we conducted a search for putative target genes by using miRanda tool and transcriptome sequene libraries of banana maintained in NCBI database. A strict selection criteria was used to identify putative miRNA targets wherein hits having minimum free energy < = -40 were considered exclusively. Using this criteria a total of 173 putative targets were predicted for selected known and novel miRNAs in *Grand Naine* (S3 Table; and S4 Table) and 161 targets were predicted for selected known and novel miRNAs in *Rasthali* (S5 Table; and S6 Table).

### Generation of transgenic banana plants

**Fig 2:**
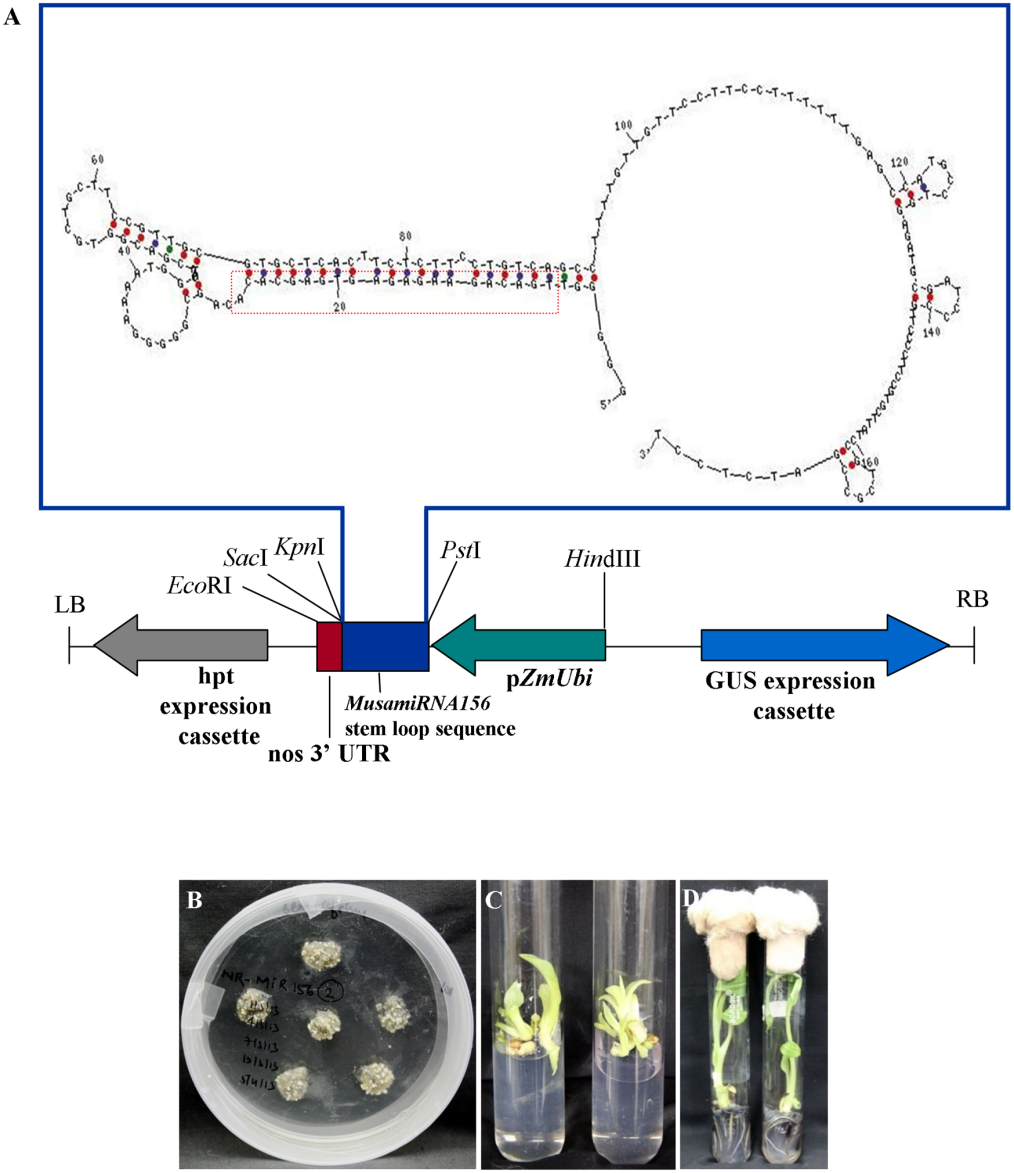
Generation of transgenic banana plants overexpressing *MusamirRNA156*. (A) Schematic representation of the T-DNA region of the binary vector p1301-*MusamiRNA156* depicting the insertion of the stem-loop sequence of *MusamirRNA156* primary transcript in the multiple cloning site of modified pCAMBIA-1301 vector downstream of *Zea mays* polyubiquitin promoter. Putatively transformed embryos selected on the medium supplemented with hygromycin (B) followed by germination. The shoots were multiplied in medium containing BAP to form the clonal copies of the shoots (C) which were further rooted in NAA based medium (D).

A 175 bp sequence encompassing the predicted stem loop region of *MusamiRNA156* gene was amplified from genomic DNA of banana cv. *Rasthali*, cloned into binary vector (Fig 2A) and was transformed into transgenic banana plants using *Agrobacterium tumefaciens* EHA105. Embryogenic cells of banana used as explants in this transformation procedure formed embryos on the medium supplemented with hygromycin (Fig 2B). These embryos were subcultured for three rounds on hygromycin supplemented selection medium. Putatively transformed embryos were then germinated and transferred to shoot multiplication medium. Multiple shoots of each transformation event obtained were later rooted in medium containing NAA (Fig 2C and 2D).

### Histochemical GUS staining

**Fig 3:**
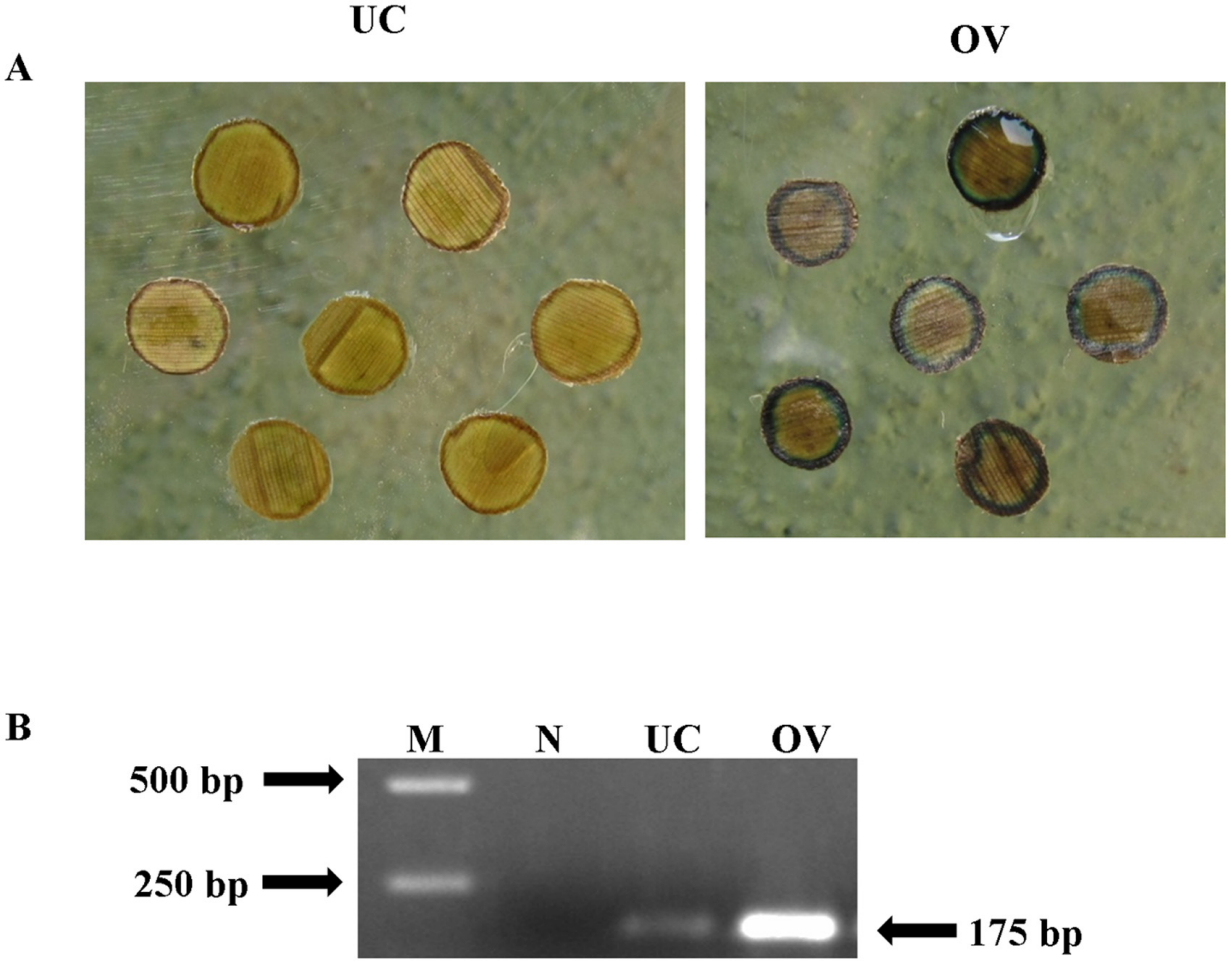
Expression analysis of the T-DNA genes in transgenic banana lines. (A) The expression of the GUS cassette in transgenic banana lines was determined by histochemical staining of the leaf discs and showed the presence of intense blue coloration in the transgenic lines (OV) whereas no colour was observed in the untransformed control plants (UC). (B) The quantum of the primary transcript of *MusamirRNA156* in the transgenic banana line and untransformed control plant was determined by RT-PCR wherein a 175 bp product was seen in both untransformed control plant and selected transgenic line with later being brighter. (M- 1kb DNA ladder; N- RT-PCR control)

The leaf discs were cut out from leaf lamina of p1301-*MusamiRNA156* derived banana plant and untransformed control banana plant were incubated overnight in GUS buffer. On fixing the leaf discs with 70% ethanol showed deep blue coloration in the leaf discs of p1301-*MusamiRNA156* derived banana plant whereas no staining was seen in the leaf discs of untransformed control banana plant (Fig 3A).

### Detection of pri-miRNA156 sequence by PCR

To investigate the expression of the pri-*MusamiRNA156* in transgenic banana plants, total RNA extracted (5 μg) from the p1301-*MusamiRNA156* derived banana plant and untransformed control banana plant was reverse transcribed into cDNA and used as template in the PCR amplification reaction. A single amplified product of 175 bp was clearly visible in both the samples (Fig 3B). The level of expression of this band in p1301-*MusamiRNA156* derived banana plant was significantly higher than in the untransformed control banana plant signifying efficient overexpression of *MusamiRNA156* in transgenic banana plants.

### Morphological analysis

**Fig 4:**
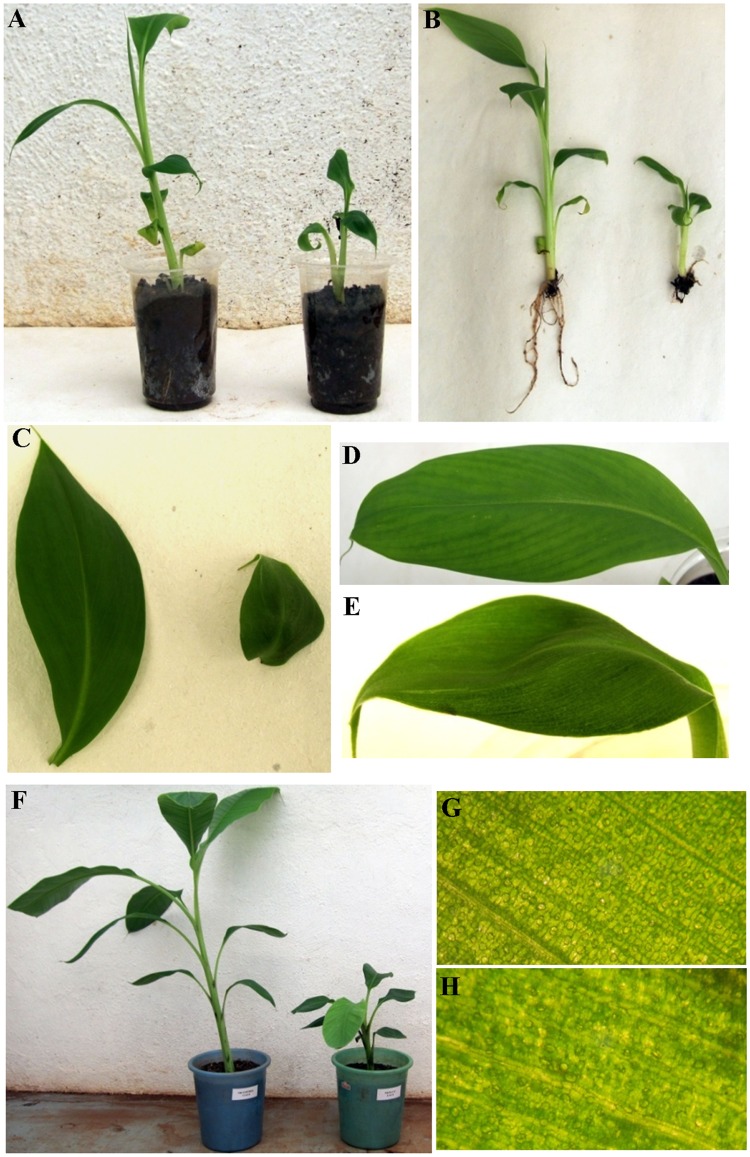
Phenotypes of the untransformed banana plant and p*MusamiRAN156*-1301 derived transgenic banana plant. (A) Gross difference in the growth of the untransformed banana plant and p1301-*MusamiRNA156* derived transgenic banana plant. (B) Poor development of the root system in the two plants 1 month post hardening. Difference in the leaf anatomy of the untransformed banana plant and p1301-*MusamiRNA156* derived transgenic banana plant (C, D and E). Growth difference seen in the untransformed banana plant and p1301-*MusamiRNA156* derived banana plant, three months post hardening (F). Microscopic examination of the leaf cellular pattern seen in the untransformed banana plant and p1301-*MusamiRNA156* derived transgenic banana plant (G and H).

In order to investigate the role of *MusamiRNA156* in the vegetative tissues of banana we undertook overexpression of *MusamiRNA156* in transgenic banana plants. These transgenic banana plants showed stunted growth as compared to untransformed controls from initial stages of growth. There was significant difference in the height of the two groups of plants under study (Fig 4A). A transgenic banana plant uprooted from soil 1 month post hardening showed poor root development (Fig 4B). The length and number of roots in the transgenic banana plant was notably lower than the untransformed control plant. Transgenic banana plants accumulating higher levels of *MusamiRNA156* showed significant difference in the leaf morphology. The width and length of the fully expanded leaf of transgenic banana plant was significantly reduced (Fig 4C). The leaves were thick and showed dark green streaks with a unique shape. Later the plants were transferred to bigger pots and maintained in green house for three months wherein the transgenic banana plant continued to show reduced height and reduced leaf length and width (Fig 4D, 4E and 4F). A simple stereomicroscope image of the abaxial side of the banana leaves derived from transgenic and control banana plants showed clear difference in the arrangement of cells in the longitudinal rows with the transgenic leaves showing more randomised pattern (Fig 4G and 4H). This difference in the morphology of leaf surface clearly indicated important roles for *MusamiRNA156* in leaf growth and development in banana.

## Discussion

MiRNAs have recently emerged as a novel class of gene regulators. Several studies over the last decade have indicated their importance in normal growth and development of plants [26]. Banana being the most important fruit crop of the world (in terms of consumption and the quantum of total production weight), understanding the functions of diverse miRNAs in regulating growth and development of banana is necessary. Since most of the edible bananas have a genomic lineage derived from the ancient *Acuminata* and *Balbisiana* genomes [27], we have carried out small RNA sequencing of two cultivars with contrasting genome composition and unique vegetative and fruit characteristics. Further, to elucidate the significance of microRNA expression in different cultivars, we overexpressed one of the most commonly studied and abundant microRNAs (*MusamiRNA156*) in transgenic banana plants. The fact that small RNA profiling of the two cultivars indicated a 5 fold difference in levels of *MusamiRNA156* in young leaves justified its overexpression in transgenic banana plants.

Among the two cultivars that we chose (for sequencing of their small RNA), cv. *Grand Naine* has three A genomes (derived from Acuminata lineage) and cv. *Rasthali* possesses two A genomes and a B genome (derived from Balbisiana lineage). We selected these two cultivars based on their elite fruit characteristics and the economic importance. Investigations into the relative profiles of major miRNAs in the two cultivars are expected to lead to better understanding of the major physiological processes in the two cultivars. Taking advantage of the newly sequenced *Acuminata* and *Balbisiana* genomes [15, 16] we could detect several conserved miRNAs in these libraries. Additionally, a number of novel miRNAs were predicted in each of the cultivar using commonly employed algorithms. Between the two cultivars approx. 44 to 53% miRNAs identified were found to unique to each cultivar. Thus, even though the two cultivars theoretically share 66% of their genome the percentage of unique miRNAs indicated significant cultivar specific evolutionary changes in miRNA identity and profiles.

Like in other such studies, the small RNA sequencing of these two cultivars showed that the 24 nt length miRNA class dominates in the dataset by a good majority [8, 9, 10]. This further indicates that in banana also small RNA directed heterochromatin silencing is an important phenomenon in gene regulation [7, 28]. In a complete contrast to these findings, when a double stranded RNA cassette is expressed in banana [29], majority of small RNAs belong to 21 and 19 nt class. This study performed in cv. *Rasthali* conclusively proves the earlier observations in other plants that unique DICER proteins process RNA hairpins with different origins [29, 30].

To extend the findings of this study, we chose to overexpress one microRNA which showed significant difference in the abundance in the two libraries. When *MusamiRNA156* was oevrexpressed in cv. *Rasthali* using its stem-loop sequence amplified from cv. *Rasthali*, we observed significant growth alterations in the transgenic lines indicating the involvement and the importance of miRNA156 in banana growth and development. The altered appearance of the transgenic lines could be seen even after 6 months of growth in a contained greenhouse. In contrast to other plants where miRNA156 has been overexpressed before, the growth penalty was not so severe in banana. This was despite the fact that we noticed strong overexpression of the *MusamiRNA156* transcript derived from the T-DNA in transgenic lines as compared to equivalent controls.

The findings of this study will help in better understanding of a myriad of physiological processes in banana. The importance of small RNA based regulation of gene functions in plants has been recognized in the last decade. This study describing the small RNA profile of two important cultivars of banana will facilitate investigations into the roles played by small RNAs in economically important banana traits like fruit ripening and disease resistance.

## Supporting Information

S1 Table Predicted conserved miRNA in *Grand Naine* and *Rasthali*. (XLS)

S2 Table Predicted novel miRNA in *Grand Naine* and *Rasthali*. (XLS)

S3 Table *Grand Naine* conserved miRNA targets. (XLS)

S4 Table *Grand Naine* novel miRNA targets. (XLS)

S5 Table *Rasthali* conserved miRNA targets. (XLS)

S6 Table *Rasthali* novel miRNA targets. (XLS)

## References

[1] Vaucheret H (2006) Post-transcriptional small RNA pathways in plants: mechanisms and regulations. Genes Dev 20: 759–771. 16600909 10.1101/gad.1410506

[2] Voinnet O (2009) Origin, biogenesis, and activity of plant MicroRNAs. Cell 136: 669–687. 10.1016/j.cell.2009.01.046 19239888

[3] Papp IM, Mette F, Aufsatz W, Daxinger L, Schauer SE, Ray A, et al (2003) Evidence for nuclear processing of plant micro RNA and short interfering RNA precursors. Plant Physiol 132: 1382–1390. 12857820 10.1104/pp.103.021980 PMC167078

[4] Khraiwesh B, Zhu JK, Zhu J (2011) Role of miRNAs and siRNAs in biotic and abiotic stress responses of plants. Biochimica. et Biophysica. Acta 1819: 137–148. 10.1016/j.bbagrm.2011.05.001 21605713 PMC3175014

[5] Zhou X, Sunkar R, Jin H, Zhu JK, Zhang W (2009) Genome-wide identification and analysis of small RNAs originated from natural antisense transcripts in *Oryza sativa* . Genome Res 19: 70–78. 10.1101/gr.084806.108 18971307 PMC2612963

[6] Jeong DH, Park S, Zhai J, Gurazada SGR, De Paoli E, Meyers BC, et al (2011) Massive analysis of rice small RNAs: mechanistic implications of regulated microRNAs and variants for differential target RNA cleavage. Plant. Cell 23: 4185–4207 10.1105/tpc.111.089045 22158467 PMC3269859

[7] Moxon S, Jing R, Szittya G, Schwach F, Pilcher RL, Moulton V, et al (2008) Deep sequencing of tomato short RNAs identifies microRNAs targeting genes involved in fruit ripening. Genome Res 18: 1602–1609. 10.1101/gr.080127.108 18653800 PMC2556272

[8] Zhao YT, Wang M, Fu SX, Yang WC, Qi CK, Wang XJ (2012) Small RNA profiling in two *Brassica napus* cultivars identifies microRNAs with oil production and development correlated expression and new small RNA classes. Plant Physiol 158: 813–823. 10.1104/pp.111.187666 22138974 PMC3271769

[9] Jia L, Zhang D, Qi X, Ma B, Xiang Z, Ningjia H (2014) Identification of the conserved and novel miRNAs in mulberry by high-throughput sequencing. PLoS ONE 9: e104409 10.1371/journal.pone.0104409 25118991 PMC4131894

[10] Zhang R, Marshall D, Bryan GJ, Hornyik C (2013) Identification and characterization of miRNA transcriptome in potato by high-throughput sequencing. PLoS ONE 8: e57233 10.1371/journal.pone.0057233 23437348 PMC3578796

[11] Wang L, Liu H, Li D, Chen H (2011) Identification and characterization of maize microRNAs involved in the very early stage of seed germination, BMC Genomics 12: 154 10.1186/1471-2164-12-154 21414237 PMC3066126

[12] Mohapatra D, Mishra S, Sutar N (2010) Banana and its byproduct utilization: An overview. J Sci Ind Res (India) 69: 323–329.

[13] Tee YK, Ding P, Rahman NAA (2011) Physical and cellular structure changes of *Rasthali* banana (*Musa* AAB) during growth and development. Sci Hortic 129: 382–389.

[14] Ghag SB, Shekhawat UKS, Ganapathi TR (2014a) Native cell-death genes as candidates for developing wilt resistance in transgenic banana plants. AoB PLANTS 10.1093/aobpla/plu037 PMC4122335 24996429

[15] D’Hont A, Denoeud F, Aury JM, Baurens FC, Carreel F, Garsmeur O, et al (2012) The banana (*Musa acuminata*) genome and the evolution of monocotyledonous plants. Nature 488: 213–217. 10.1038/nature11241 22801500

[16] Davey MW, Gudimella R, Harikrishna JA, Sin LW, Khalid N, Keulemans J (2013) A draft *Musa balbisiana* genome sequence for molecular genetics in polyploidy, inter- and intra-specific *Musa* hybrids. BMC Genomics 14: 683 10.1186/1471-2164-14-683 24094114 PMC3852598

[17] Xie K, Shen J, Hou X, Yao J, Li X, Xiao J, et al (2012) Gradual increase of miR156 regulates temporal expression changes of numerous genes during leaf development in rice. Plant. Physiol 158: 1382–1394. 10.1104/pp.111.190488 22271747 PMC3291253

[18] Huijser P, Schmid M (2011) The control of developmental phase transitions in plants. Development 138: 4117–4129. 10.1242/dev.063511 21896627

[19] Yu N, Cai WJ, Wang S, Shan CM, Wang LJ, Chen XY (2010) Temporal control of trichome distribution by microRNA156-targeted *SPL* genes in *Arabidopsis thaliana* . Plant Cell 22: 2322–2335. 10.1105/tpc.109.072579 20622149 PMC2929091

[20] Xing S, Salinas M, Hohmann S, Berndtgen R, Huijser P (2010) miR156-targeted and non targeted SBP-box transcription factors act in concert to secure male fertility in *Arabidopsis* . Plant Cell 22: 3935–3950. 10.1105/tpc.110.079343 21177480 PMC3027167

[21] Gou JY, Felippes FF, Liu CJ, Weigel D, Wang JW (2011) Negative regulation of anthocyanin biosynthesis in *Arabidopsis* by a miR156-targeted SPL transcription factor. Plant Cell 23: 1512–1522. 10.1105/tpc.111.084525 21487097 PMC3101539

[22] Griffiths-Jones S, Grocock RJ, van Dongen S, Bateman A, Enright AJ (2005) miRBase: microRNA sequences, targets and gene nomenclature. Nucleic Acids Res 34: D140–D144. 16381832 10.1093/nar/gkj112 PMC1347474

[23] Ganapathi TR, Higgs NS, Balint-Kurti PJ, Arntzen CJ, May GD, Van Eck JM, (2001) *Agrobacterium*-mediated transformation of embryogenic cell suspensions of the banana cultivar *Rasthali* (AAB). Plant Cell Rep 20: 157–162. 30759903 10.1007/s002990000287

[24] Ghag SB, Shekhawat UKS, Ganapathi TR (2012) *Petunia* floral defensins with unique prodomains as novel candidates for development of Fusarium wilt resistance in transgenic banana plants. PLoS ONE 7: e39557 10.1371/journal.pone.0039557 22745785 PMC3382125

[25] Ghosh A, Shekhawat UKS, Ganapathi TR, Bapat VA (2012) Analysis of banana fruit-specific promoters using transient expression in embryogenic cells of banana cultivar Robusta (AAA Group). J Plant Biochem Biot 21: 189–197.

[26] Jones-Rhoades MW, Bartel DP, Bartel B (2006) MicroRNAs and their regulatory roles in plants. Annu Rev Plant Biol 57: 19–53. 16669754 10.1146/annurev.arplant.57.032905.105218

[27] Lescot M, Piffanelli P, Ciampi AY, Ruiz M, Blanc G, Leebens-Mack J, et al (2008) Insights into the *Musa* genome: syntenic relationships to rice and between *Musa* species. BMC Genomics 9: 58 10.1186/1471-2164-9-58 18234080 PMC2270835

[28] Fahlgren N, Howell MD, Kasschau KD, Chapman EJ, Sullivan CM, Cumbie JS, et al (2007) High-throughput sequencing of *Arabidopsis* microRNAs: evidence for frequent birth and death of *MIRNA* genes. PLoS ONE 2: e219 17299599 10.1371/journal.pone.0000219 PMC1790633

[29] Ghag SB, Shekhawat UKS, Ganapathi TR (2014b) Host induced post-transcriptional hairpin RNA-mediated gene silencing of vital fungal genes confers efficient resistance against Fusarium wilt in banana. Plant Biotechnol J 12: 541–553. 10.1111/pbi.12158 24476152

[30] Liu Y, Wang M, Wang X (2014) Endogenous small RNA clusters in plants. Genomics Proteomics Bioinformatics 12: 64–71. 10.1016/j.gpb.2014.04.003 24769055 PMC4411336

